# Creating Tunable Quantum Corrals on a Rashba Surface Alloy

**DOI:** 10.1021/acsnano.2c00467

**Published:** 2022-03-10

**Authors:** Wouter Jolie, Tzu-Chao Hung, Lorena Niggli, Benjamin Verlhac, Nadine Hauptmann, Daniel Wegner, Alexander Ako Khajetoorians

**Affiliations:** Institute for Molecules and Materials, Radboud University, 6525 AJ Nijmegen, The Netherlands

**Keywords:** Rashba effect, artificial lattices, scanning tunneling microscopy, quantum corral, warping, spin−orbit interaction

## Abstract

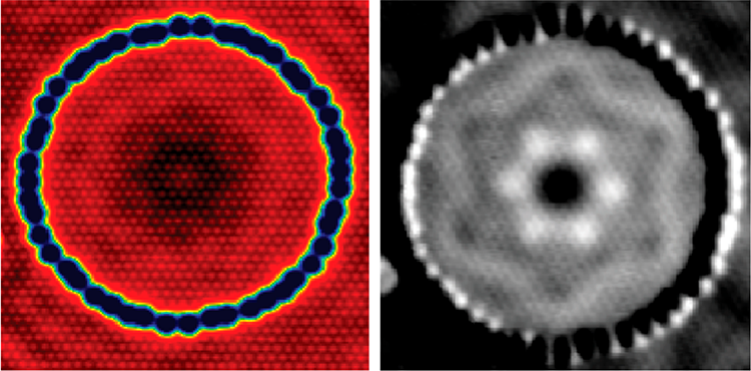

Artificial lattices derived from assembled atoms on a surface using scanning tunneling microscopy present a platform to create matter with tailored electronic, magnetic, and topological properties. However, artificial lattice studies to date have focused exclusively on surfaces with weak spin–orbit coupling. Here, we illustrate the creation and characterization of quantum corrals from iron atoms on the prototypical Rashba surface alloy BiCu_2_, using low-temperature scanning tunneling microscopy and spectroscopy. We observe very complex interference patterns that result from the interplay of the size of the confinement potential, the intricate multiband scattering, and hexagonal warping from the underlying band structure. On the basis of a particle-in-a-box model that accounts for the observed multiband scattering, we qualitatively link the resultant confined wave functions with the contributions of the various scattering channels. On the basis of these results, we studied the coupling of two quantum corrals and the effect of the underlying warping toward the creation of artificial dimer states. This platform may provide a perspective toward the creation of correlated artificial lattices with nontrivial topology.

Understanding the interplay of topology, spin–orbit coupling, and electron-mediated interactions in crystal structures and how they relate to various quantum phases of matter is one of the central goals in condensed matter physics. There are many approaches in recent years that have been developed to create designer matter, where tailored electronic or magnetic structures are created by bottom-up approaches.^1−4^ Of the variety of approaches available today, scanning tunneling microscopy and spectroscopy (STM/STS) provide a powerful toolbox combining atomic-scale fabrication and on-site characterization.^4,5^ In this paradigm, impurities are patterned on a surface using atomic manipulation, and the development of tailored properties is monitored with STM/STS. For example, artificial lattices have been constructed to realize lower dimensional band structures,^6−9^ topological edge states,^10^ and synthetic Dirac quasiparticles,^5,11,12^ as well as serve as a platform for topological superconductivity.^13,14^

Quasiparticle interference (QPI) is an important aspect in creating artificial lattices based on the STM/STS approach. This is exemplified by the pioneering example of the quantum corral (QC),^15,16^ where strong interference leading to atomic-like states was created by sculpting the scattering potential stemming from impurity-induced QPI. The confinement potential of QCs made in this fashion has been utilized to sculpt the Kondo effect via the quantum mirage,^17^ as well as used to create coupled artificial atoms.^16,18^ More recently, this concept was expanded to create periodic lattices of coupled artificial atomic sites yielding synthetic band structures, as exemplified by the creation of molecular graphene.^5^ In this approach, patterned impurities lead to periodic scattering, where focused quasiparticles weakly interact between chosen artificial sites on the surface.^19^ Nevertheless, these experiments have focused exclusively on single band scattering using the QPI originating from the surface state of Cu(111), which is isotropic and exhibits weak spin–orbit coupling.^20^ The synthetic development of many classes of electronic structure requires incorporating strong spin–orbit coupling in these platforms.^21^ This necessitates the development of high-quality surfaces, which exhibit large spin–orbit coupling and strong QPI, suitable for atomic manipulation, as well as understanding the role of multiband scattering in creating artificial lattice sites.

Here, we tailored artificial QCs, derived from the QPI originating from electronic bands of the Rashba surface alloy BiCu_2_ grown on Cu(111).^22,23^ Using STM/STS, we first characterized the structural and electronic properties of individual Fe atoms. Using atomic manipulation, we then created QCs of various sizes. On the basis of spatially dependent measurements, we observed strongly anisotropic features at particular energies, which deviate from QCs that we fabricated from CO on Cu(111) and depend strongly on the size of the QC. Using a particle-in-a-box model and comparing to spatially dependent spectroscopy, we traced the rich behavior of the confined wave functions to the interplay between multiband scattering, hexagonal warping, and the size of the QC. Finally, we constructed coupled QC pairs that take advantage of the warping behavior of the scattered quasiparticles on the BiCu_2_ platform, and we illustrate wave functions that show weak coupling between the two QCs via incipient bonding–antibonding splitting.

Upon deposition of Bi on Cu(111) and annealing (cf. methods for further experimental details), Bi atoms embed in the topmost Cu(111) layer and form a well-ordered  superstructure (BiCu_2_).^24^ The surface alloy can be imaged using STM constant-current imaging (Figure 1a). BiCu_2_ features two strongly confined, hole-like surface states, which exhibit a giant Rashba effect due to large spin–orbit coupling.^25−27^ While the inner band is almost isotropic, the outer band shows pronounced hexagonal anisotropy, leading to increased nesting along specific directions in momentum space. This can be directly imaged using QPI (see also Figure S2) as well as with angular resolved photoemission.^22,23,25−27^ A sketch of the band structure is depicted in Figure 1d,e, including the possible spin-conserving QPI scattering vectors.

**Figure 1:**
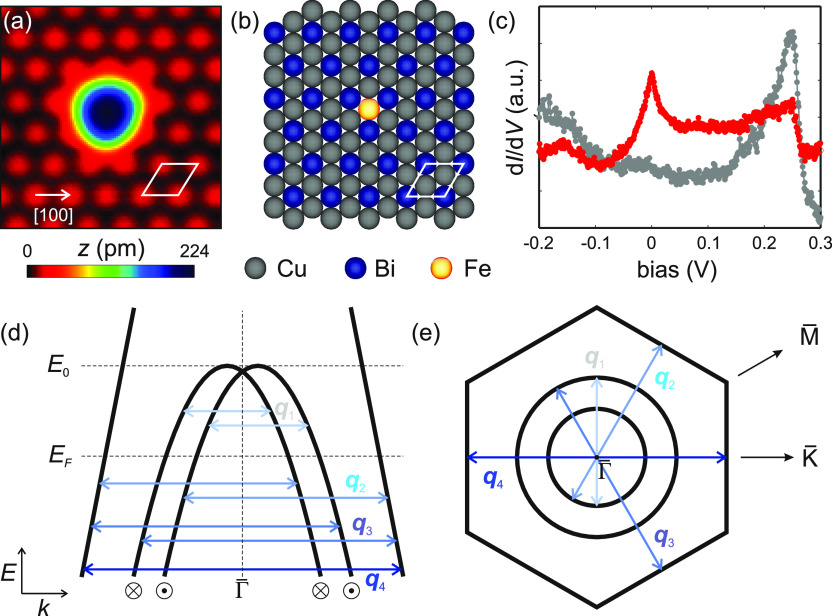
Single Fe atom on BiCu_2_. (a) Constant-current STM image of a single Fe atom on BiCu_2_ (*V*_s_ = 5 mV, set point current *I*_t_ = 100 pA). (b) Structural model of (a), revealing the adsorption site of Fe. (c) Point spectra taken on BiCu_2_ (gray) and Fe (red), respectively (*V*_stab_ = −0.2 V, *I*_stab_ = 100 pA). (d) Sketch of the hole-like surface states of BiCu_2_. The Fermi energy *E*_F_ and onset *E*_0_ of the inner Rashba-split band are denoted. Blue colored arrows represent intraband (**q**_1_ and **q**_4_) and interband (**q**_2_ and **q**_3_) scattering vectors, which lead to visible quasiparticle interference. (e) Simplified cartoon of the constant-energy contour of BiCu_2_ in momentum space. The inner Rashba-split band shows circular symmetry, while the outer band shows hexagonal symmetry.

## Results and Discussion

### Individual Fe Atoms and the BiCu_2_ Surface

In the subsequent experiments, we created large terraces of high-quality BiCu_2_, followed by deposition of individual Fe atoms on the surface to create well-defined point scatterers. Fe atoms appear in constant-current imaging (Figure 1a) as a round-like protrusion. By imaging the surrounding lattice, we found that Fe preferably adsorbs on top of Cu atoms, as depicted in the corresponding structural model in Figure 1b. Fe is surrounded by three Bi atoms, leading to two equivalent adsorption sites with three-fold symmetry and to an apparent triangular deformation from the round shape of the Fe atom (Figure 1a). We measured point spectra (d*I*/d*V*) of individual Fe atoms, in comparison to the BiCu_2_ substrate, as illustrated in Figure 1c. Spectra of bare BiCu_2_ exhibit a pronounced peak caused by a van Hove singularity at a sample voltage of *V*_s_ = 0.25 V, resulting from the onset of the inner Rashba-split *sp*_*z*_ band at *E*_0_, as the band maxima are shifted away from the Γ̅ point (Figure 1d).^23,28^ In comparison, individual Fe atoms are rather featureless in the probed energy window, aside from a pronounced Fano-like resonance at the Fermi energy. While this feature is not the focus of this manuscript, it may be attributed to a Kondo resonance,^15,29^ with a half-width at half-maximum (HWHM) of roughly Γ = 10 mV. In the subsequent discussion, we consider the Fe atoms as hard-wall scatterers and neglect the contributions to the QPI induced by the Kondo-like feature. We normalized all spectra taken in the generated QCs by dividing them with the substrate reference spectrum.

### Single Quantum Corrals

**Figure 2:**
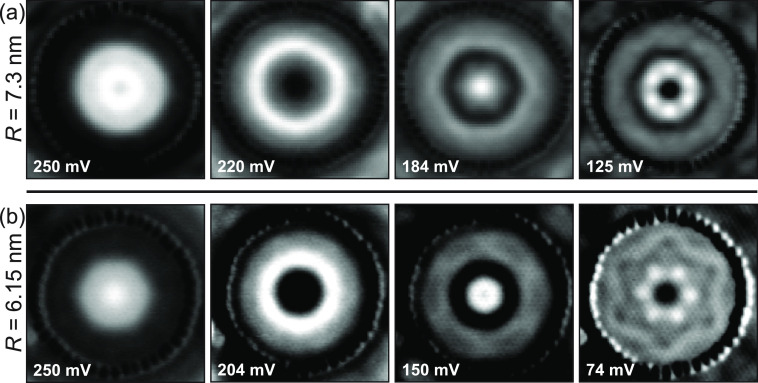
Confined states of the Rashba QC with tunable anisotropy for two different radii. (a) Real-space constant-current d*I*/d*V* maps of a QC with *R* = 7.3 nm built from 56 Fe atoms. The superposition with eigenstates of the outer band as well as interband eigenstates creates patterns with clear hexagonal symmetry (**q**_2_–**q**_4_). (b) Real-space constant-current d*I*/d*V* maps of the QC with *R* = 6.15 nm built from 48 Fe atoms. *I*_t_ = 100 pA (a) and 200 pA (b). Standard linear grayscale mapping was used for all images and adjusted individually to maximize contrast.

In Figure 2, we detail constant-current imaging of the local density of states (LDOS) at various selected energies of two circular QCs with different radii by d*I*/d*V* maps (*R* = 7.3 nm (a) and 6.15 nm (b); additional maps can be found in Figure S5). As Fe atoms exhibit strong QPI, the formation of the QC leads to a strong quantum confinement potential. The patterns imaged at a given energy inside the QC are related to the interference of standing waves originating from the various scattering channels (*q*_i_) in the band structure (Figure 1d–e; see also Figure S2). Reducing the radius of the QC pushes the energy of the confined states further away from the onset energy of the surface state, as expected from a particle-in-a-box model. In the original quantum corral work by Crommie et al.,^15^ this led to circular fringes at all energies, stemming from a single isotropic scattering channel, related to the dispersion of the Shockley surface state of Cu(111). In comparison here, more complex patterns were observed within a given QC, depending on the radius and *V*_s_. For *R* = 7.3 nm (Figure 2a), we found circular-like fringes near the onset of the inner band. Going lower in energy (i.e., following the downward dispersion), there was a strong deviation from circular fringes, exemplified by the emergence of hexagonal patterns within the circular confinement potential featuring a complex spatial distribution within the QC. Intriguingly, we also found that some of the multiple hexagonal patterns at a given energy are rotated by 30° with respect to each other (e.g., *V*_s_ = 125 mV). In comparison, the QC with radius *R* = 6.15 nm exhibited noticeably different distributions, compared to the previous QC. The emergence of hexagon patterns can be seen closer to the band onset, whereas the interplay of different hexagonal fringes with different apparent symmetry, compared to the *R* = 7.3 nm QC, leads to vastly different wave functions at energies further away from the band onset. We emphasize that the QCs are almost perfectly circular rings (see structural models in Figure S12); hence the scattering potential is circularly symmetric and cannot account for the observation of hexagonal QPI features. We also verified that the observed constant-current d*I*/d*V* maps did not produce artifacts, by comparison with constant-height measurements (cf. Supporting Information section 7, Figures S7–S9).

In order to understand the origins of these patterns, we first revisit the possible scattering vectors stemming from the BiCu_2_ band structure.^23^ At a given energy, the QPI patterns are dominated by a set of scattering wave vectors that connect two states in the band structure (Figure 1d). Here, we assume the outer *p*_*x*_*p*_*y*_ band (with large wave vectors) to be spin-degenerate close to the Fermi energy, in line with angular-resolved photoemission measurements.^22,25^ In contrast, the inner band is strongly spin-split due to the Rashba effect. This leads to four possible spin-conserving scattering wave vectors: two intraband scattering wave vectors **q**_1_ and **q**_4_, which connect states within the same band, and two interband scattering wave vectors **q**_2_ and **q**_3_ that connect states between inner and outer bands, respectively. The **k**-dependence of these scattering wave vectors at a fixed energy is shown in Figure 1e, which displays a constant-energy contour of the band structure in momentum space. While **q**_1_ is isotropic, **q**_2_–**q**_4_ inherit the hexagonal warping of the outer band. We verified that the dispersions and anisotropies of **q**_1_ to **q**_4_ within the QCs are in agreement with results from QPI of the bare BiCu_2_ surface;^23^ i.e., the underlying band structure of the quantum-confined states is unchanged within the QCs (see Supporting Information section 3, Figure S2).

**Figure 3:**
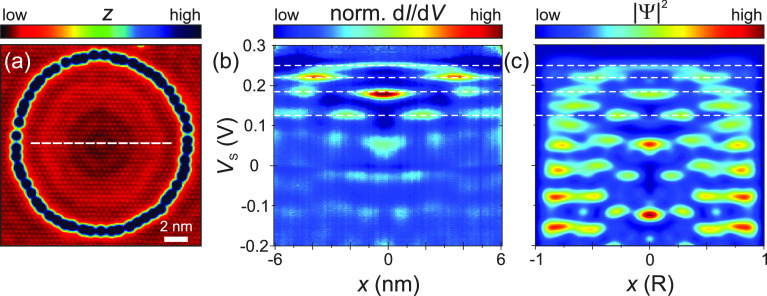
Dispersion of a Rashba QC. (a) Constant-current STM image of a QC built from 56 Fe atoms with *R* = 7.3 nm (*V*_s_ = 5 mV, *I*_t_ = 100 pA). (b) Set of normalized STS spectra measured along the cross-sectional line indicated in (a) (*V*_stab_ = −0.2 V, *I*_stab_ = 200 pA). (c) Particle-in-a-box simulation of the QC, containing contributions from **q**_1_, **q**_2_, and **q**_3_ with fixed weights (*w*(**q**_1_) = 5*w*(**q**_2_) = 5*w*(**q**_3_)). The horizontal dashed lines denote the energies of the maps shown in Figure 2a and Figure S5a.

We subsequently measured d*I*/d*V* point spectra along the diameter of a QC (Figure 3). Each spectrum was taken after moving to a subsequent point along the line, with the feedback loop enabled at large bias voltage leading to an effective constant height for all positions within the QC (see Supporting Information section 7), and then measuring the point spectrum with the feedback loop opened at the stabilization voltage. Figure 3b shows the resulting normalized d*I*/d*V* intensity in a false-color plot as a function of *V*_s_ and position along the diameter. The regions of high intensity can be associated with the LDOS of the localized wave functions (|ψ|^2^) along the cross section of the QC, which are related to the various states of the particle in a box and are qualitatively in good agreement with the observed spatial maps (cf. Supporting Information section 7). The data reveal a discrete set of standing waves inside the QC, starting with the first eigenstate close to the band maximum of the inner Rashba-split band and with the wavelength of the higher states decreasing with decreasing energy, as expected for hole-like bands.

To understand the interplay between the confined wave functions and the various band contributions, we simulated the confined wave functions using a circular particle-in-a-box approach (see Supporting Information section 3). For BiCu_2_, the presence of multiple scattering wave vectors necessitates considering more channels, each with a given contribution *w*(**q**_i_) to the LDOS. We considered the four scattering wave vectors **q**_1_–**q**_4_ to account for all the features observed in the experiments (Figure S2), and treated each of them in an independent particle-in-a-box problem, respectively (Figure S3). We simplified the confinement potential generated by individual Fe atoms using an infinite potential. In order to account for the finite size of each Fe atom, we considered a circular potential of the QC with a theoretical radius reduced by the atomic radius of Fe (0.15 nm). The solutions of the Schrödinger equation for the circular potential are *l*th order Bessel functions, *J*_*l*_, defined by a set of quantum numbers (*n*,*l*) with momentum *k*_*n*,*l*_ = *z*_*n*,*l*_/*R* and energy , with m* the effective mass of the nearly free surface band and *z*_*n*,*l*_ the *n*th zero crossing of *J*_*l*_(*z*).^15^ The resultant LDOS is given by , where *N*_*n*,*l*_ ensures proper normalization. To be able to simulate the various wave vectors using a free-particle Hamiltonian, we analytically described the dispersions of all four scattering wave vectors using parabolic fits of the underlying bands to obtain the onset energies *E*_0,**q**_ and effective masses . We note that this approach cannot account for the radial anisotropy of the band structure (see Supporting Information section 3 for a detailed discussion). This results in a set of wave functions (*n*,*l*)_**q**_ for all four confined scattering wave vectors. To verify the particle-in-a-box model, we measured the situation for a QC built from CO molecules on Cu(111) and simulated it using a single isotropic scattering channel based on the Cu(111) surface state (Figure S1). In this case, we found reasonable agreement between the experimental results and the model.

The resulting simulation for the QC with *R* = 7.3 nm is plotted in Figure 3c. On the basis of previous QPI measurements from native defects,^23^ the relative strength of each scattering channel (**q**_i_) may vary. We therefore manually weighed the contribution of all four channels approximately, using experimental input, and found reasonable qualitative reproducibility at particular energies when using *w*(**q**_1_) = 5*w*(**q**_2_) = 5*w*(**q**_3_) (Figure S3). We note that **q**_3_ contributions have not been observed before in QPI of native defects^23^ but were relevant to describe these experiments. This most likely stems from differences in the scattering potential between native defects and individual Fe atoms.

We find that most of the intensity in the unoccupied states stems from the isotropic inner band (**q**_1_). Nevertheless, there are additional contributions from **q**_2_ and **q**_3_, leading to the deviations from circular fringes. For instance, the position-dependent energy and broadening of the state near *V*_s_ = 50 mV in the center of the QC are found both experimentally and theoretically and can be traced back to a local superposition of **q**_1_ and **q**_3_ states (further fingerprints of **q**_3_ are discussed in Supporting Information section 4, Figure S3). Similarly, a wealth of additional modes is found in the simulation near the rim.^30^ We note that the addition of **q**_4_ contributions did not lead to further improvement of the agreement between experiment and model (Figure S4). While the inclusion of interband scattering (**q**_2_ and **q**_3_) helps to improve the comparability between simulation and experiment for the unoccupied states, there are more qualitative differences in the occupied region. For instance, the alternation between nodes and antinodes in the center of the QC from one confinement state to the next, as expected from a particle-in-a-box model and indeed found in our simulation, is not followed in the experiment when crossing the Fermi level: the confined states seen near *V*_s_ = 50 mV and *V*_s_ = −25 mV both have antinodes. d*I*/d*V* maps at these energies (Figure S14) show that these states are indeed two qualitatively different states. This difference between model and experiment may result from the assumption of a constant infinite scattering potential, which may be too simplified. Recent QPI measurement on Re(0001) revealed a sign change of the scattering potential when crossing *E*_F_, which was attributed to a different scattering behavior of holes and electrons.^31^ In contrast, measurements of the Ag(111) surface state showed no sign change at *E*_F_,^32^ in agreement with our measurements of QCs on bare Cu(111) (see Supporting Information section 2, Figure S1). In addition to a change in sign, hybridization of the Fe atomic states with the substrate may also create an energy-dependent scattering potential, which may effectively absorb and/or transmit part of the scattered waves. We note that at larger negative energy, deeper into the occupied bands, we found a tendency toward more uniform hexagonal type patterns in the QC (Figure S5). We also considered QCs with different radii, both experimentally and theoretically (Figure S6).

Tailoring periodic arrays of atomic scatterers can be utilized to create artificial lattices.^5,10,12,19,33−35^ Within this paradigm, a periodic array of scatterers is used to create an antilattice, which focuses quasiparticles at periodic positions leading to an artificial lattice site. This strategy often depends on isotropic scattering stemming from a single scattering channel and neglects additional effects such as bulk scattering or energy-dependent potential scattering from the impurities. By use of BiCu_2_ as a prototypical Rashba platform, the design of artificial lattices is complicated by the compilation of effects demonstrated above: anisotropic scattering, a complex scattering potential, and the presence of multiple scattering channels. For example, the presence of multiband scattering will ultimately delocalize artificial lattice sites, due to the different modulations present, as seen in Figure 2.

### Coupled Quantum Corrals

**Figure 4:**
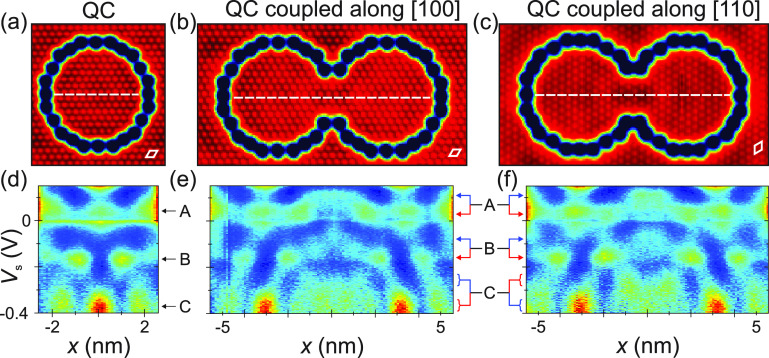
Anisotropic coupling of Rashba QC pairs. (a) STM constant-current image of a small (*R* = 3.1 nm) QC (*V*_s_ = 5 mV, *I*_t_ = 20 pA). (b) STM image of a QC pair coupled along [100] (*V*_s_ = 5 mV, *I*_t_ = 20 pA). (c) STM image of a QC pair coupled along [110] (*V*_s_ = 10 mV, *I*_t_ = 100 pA). (d–f) Set of normalized spectra measured along the line denoted in (a)–(c), respectively (*I*_stab_ = 200 pA, *V*_stab_ = −0.4 V). Three QC states (labeled A, B, and C) are marked by black arrows in (d). In the QC pairs, these states split into bonding (blue arrows) and antibonding (red arrows) states, respectively, as shown in the maps in Figure 5. A set of spectra displaying the full energy range probed can be found in Figure S10.

In order to understand how to couple artificial atoms in this vain, we created QC pair structures to emulate a dimer. The building block is a QC with *R* = 3.1 nm built from 24 Fe atoms (Figure 4a, structural model shown in Figure S13), connected via a weak link in the structure to induce a wave function overlap.^18^ We removed three Fe atoms at one side of the circular QC and fused a second constructed QC of same size next to it. This way, we constructed two nearly equivalent QC pairs, which are rotated by 30° with respect to each other (Figure 4b,c), and we compare their respective electronic properties to each other as well as to the isolated QC.

**Figure 5:**
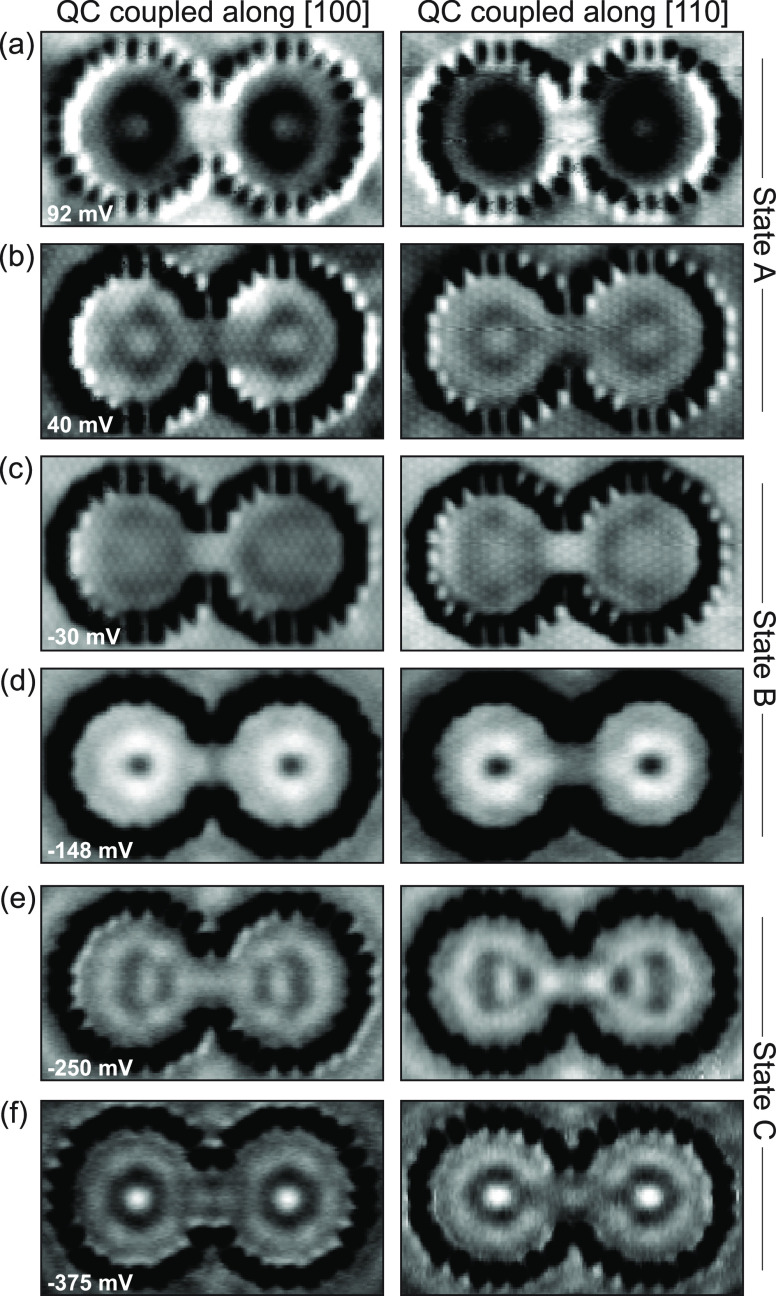
Anisotropic coupling in Rashba QC pairs. Constant-current d*I*/d*V* maps of the QC pair coupled along [100] (left column) and along [110] (right column), taken at the respective bonding and antibonding state voltages of states A–C, as identified in Figure 4 (*I*_t_ = 200 pA). The spatial LDOS distributions confirm the assignments, with the bonding (antibonding) states displaying an antinodal (nodal) LDOS at the center of the QC pair. Differences in the spatial LDOS distributions for [100] vs [110] coupled QC pairs reveal anisotropic (i.e., orientation-dependent) coupling.

Starting with the single QC, we measured STS spectra along a line as done for the previous QCs (Figure 4a). The corresponding position- and energy-dependent normalized d*I*/d*V* intensity is shown in Figure 4d (see Figure S10 for the full energy range). Similar to the larger QCs in Figure 3, we found a set of resonances, but now their energy spacing is larger due to the smaller radius (a corresponding model simulation is shown in Figure S6c). Focusing on the range *V*_s_ < 0.15 V, we identified three dominating QC states that appear as peaks in the d*I*/d*V* spectra inside the QC, labeled A (located at *V*_s_ ≈ 0.05 V), B (*V*_s_ ≈ −0.15 V), and C (*V*_s_ ≈ −0.35 V), respectively (marked by arrows in Figure 4d). We compare these spectra with the results obtained across the QC pair coupled along the BiCu_2_ [100] direction (Figure 4e). Each of the states splits into two states with spatial distributions along the cross section reminiscent of bonding (blue arrow) and antibonding (red arrow) states, respectively. This splitting is difficult to identify from a single spectrum, due to the hybridization broadening of the states. However, when spatially imaging these states, we observed a node/antinode structure that is reminiscent of bonding/antibonding states (Figure 5), which we discuss below. By comparing spectra taken at the center vs the outer regions of the QC pair (see Figure S11), we identified peak positions corresponding to the bonding and antibonding states, respectively. For state A, these are found at *V*_s_ ≈ 0.12 V and *V*_s_ ≈ 0.04 V, respectively. The fact that the bonding state is at higher energy is due to the hole-like character with negative effective mass of the underlying surface bands. Likewise, we found state B to split into bonding/antibonding states at *V*_s_ ≈ −0.08 V and *V*_s_ ≈ −0.16 V, respectively. We note that the splitting is rather small compared to the widths of the states of approximately 50–70 mV (fwhm), i.e., the coupling strength is relatively small. A consequence of this incipient splitting is that bonding and antibonding states still overlap substantially, very different from the behavior of real atomic dimers where the bonding and antibonding wave functions are well-defined separate solutions of the Schrödinger equation.

To verify the above assignments and illustrate the bonding/antibonding states, we spatially mapped the LDOS at the energies where we identified the (anti)bonding states, shown in Figure 5. For state A, the map taken at *V*_s_ = 92 mV (Figure 5a) reveals a strong antinode at the center of the QC pair, while a faint node is visible in the map at *V*_s_ = 40 mV (Figure 5b). Within each of the QC pairs, the spatial distribution is qualitatively identical for both energies. This is the hallmark of a state splitting into bonding and antibonding hybrid states. For state B, we observed the same behavior for maps taken at *V*_s_ = −30 mV (Figure 5c) and *V*_s_ = −148 mV (d), respectively. We note that for state C, along the [100] direction we were not able to clearly identify bonding/antibonding splitting from the d*I*/d*V* spectra, but LDOS maps taken in a range from *V*_s_ = −0.2 to −0.3 V reveal a bonding character of the LDOS distribution (antinode at the junction, Figure 5e), while maps taken in the range −0.3 V to −0.4 V show a node at the center of the junction (Figure 5f). From this, we conclude that also this state splits upon coupling of the QCs, with a splitting energy on the same order of magnitude as the other states.

Finally, we address the orientation dependence of the coupling between the two QCs. As discussed above, the superposition of isotropic and hexagonal standing waves in BiCu_2_ leads to anisotropic patterns in isolated QCs. This is also found in d*I*/d*V* maps of the *R* = 3.1 nm QC (see Figures S6g–i, S8d–f, and S9c). We found strong evidence for a resulting anisotropic coupling when comparing d*I*/d*V* maps of QC pairs coupled along [100] (Figure 5, left column) vs [110] (right column). This effect is least obvious for state A, where the main difference is that the antinode (a) is slightly more delocalized along [100] and the node (b) is more pronounced. For state B, comparing the maps at −148 mV (d), the node along [110] is spatially much more extended than along [100]. The most severe impact of anisotropic coupling is found for state C: maps of the QC pair coupled along [110] show an antinode in the junction between −0.2 and −0.3 V (e) and a node between −0.3 and −0.4 V (f); i.e., the situation is reversed compared to the QC pair coupled along [100].

## Conclusions

We constructed Rashba-type artificial quantum corrals using atomic manipulation of Fe atoms to form circular confinement potentials on the BiCu_2_/Cu(111) surface alloy. Scanning tunneling spectroscopy revealed anisotropic multiband QPI; i.e., the spatial distribution of the wave functions was found to be much more complex when compared to QCs fabricated on surfaces with isotropic single-band scattering. We investigated the effects of hexagonal warping and multiband scattering as a function of energy for QCs with various radii. The observed complicated spatial distributions of the wave functions could be partially captured by a nearly free particle-in-a-box model considering the underlying band structure. There are clear discrepancies, particularly at lower energies in the occupied states, requiring a more detailed theoretical treatment, for example, by considering the energy-dependent scattering potential of the Fe atoms, the role of spin–orbit coupling, and the energy-dependent variations in the quasiparticle scattering. Due to the complexity of the scattering in this system and therefore the sophisticated nature of how to design an antilattice for artificial lattices, we considered how to couple QCs using this platform by constructing dimers of quantum corrals. We demonstrated that coupled states can be identified in QC pair structures, revealing anisotropic coupling. These results serve as the basis for understanding how to incorporate Rashba-type coupling for artificial lattices constructed on surfaces, including the role of multiband scattering and hexagonal warping. However, our results also showcase the limits of creating artificial lattices based on using quasiparticles from metallic substrates. Owing to the effective screening by and hybridization with the substrate, only relatively weak coupling strengths can be achieved, and the artificial dimer states overlap significantly. Moreover, in this way, it is difficult to construct artificial orbitals which are strongly localized. This is different from dimers made from real atoms and calls for exploring platforms with strongly suppressed bulk conductivity.^36,37^

## Methods

The experiments were performed in ultrahigh vacuum (UHV) using a commercial UHV LT-SPM system (Createc). A Cu(111) single crystal was repeatedly cleaned with ion bombardment (neon, 1.5 keV) and subsequently annealed to 450 °C for 10 min. Bi was evaporated on the Cu(111) surface held at 100 °C, followed by an annealing step of 10 min at 150 °C. This procedure led to large, clean terraces of the BiCu_2_ surface alloy. Single Fe atoms were deposited on the surface held at 8 K inside the cryogenic STM. All STM/STS measurements were performed at 5 K. Atomic manipulation of Fe atoms was performed with a tunneling current of ∼30 nA and a bias voltage of 5 mV. For scanning tunneling spectroscopy (STS), we use a lock-in technique with a modulation of 1–2 mV for point spectra and 2–10 mV for d*I*/d*V* maps, with a frequency of 433–777 Hz.

## Supporting Information Available

Further experimental data; computational details and analysis; QCs on Cu(111); Fourier analysis of scattering wave vectors; particle-in-a-box modeling; disentangling scattering wave vector contributions; additional maps, spectra, and modeling of QCs; ruling out artifacts in STS; additional data on coupled QC pairs; structural models of the QCs (PDF)
