# Supplementary material for: Creating Tunable Quantum Corrals on a Rashba Surface Alloy
Source: ACS Nano. 2022 Mar 10;16(3):4876–83. doi: 10.1021/acsnano.2c00467 (PMC8945344; doi:10.1021/acsnano.2c00467)
Supplement: Supplementary file 1 — nn2c00467_si_001.pdf [file nn2c00467_si_001.pdf]

Supporting information: Creating tunable quantum corrals on a Rashba surface alloy

Wouter Jolie,[1] Tzu-Chao Hung,[1] Lorena Niggli,[1] Benjamin Verlhac,[1] Nadine Hauptmann,[1] Daniel Wegner,[1,*] Alexander Ako Khajetoorians[1,*]

[1] Institute for Molecules and Materials, Radboud University, 6525 AJ Nijmegen, the Netherlands

** corresponding authors: a.khajetoorians@science.ru.nl, d.wegner@science.ru.nl*

1. Quantum corrals made of CO on Cu(111)

To test our particle-in-a-box model, we built two quantum corrals (QCs) on the Cu(111) surface using CO molecules, similar to those from Ref. 1 and to QCs built from Fe atoms on Cu(111).[2,3] The results are shown in Fig. S1. The corresponding experimentally recorded confined states are shown in Fig. S1(b,e). As expected for a particle-in-a-box model for an isotropic QC, we found an increase of the number of nodes and antinodes, as well as an alternation between nodes and antinodes in the center of the QC from one confinement state to the next, with no change when crossing E_F. For the simulation, we used a single, electron-like band which was manually fit to the experimental dI/dV signal. The theoretical radius was reduced by 0.25 nm to account for the finite size of the CO molecules.[4] In line with the experiments, we found an alternating sequence of maxima and minima in the center of the QC. Overall, the agreement between experimental and simulated results is strong in all energy ranges probed, in contrast to what was observed in the main manuscript for the QCs built on $BiCu_2$. For the smaller QC, the most obvious discrepancy is that the eigenstates are broader. We attribute this to the finite scattering potential of the CO molecules.

2. Fourier analysis of scattering wave vectors and approximated band structure of $BiCu_2$

Standing waves in $BiCu_2$ result from four possible scattering vectors ($\boldsymbol{q}_i$), based on the band structure as schematically shown in Fig. 1(d).[5] To verify that they persist also within the QCs and that the dispersion relations are hence unaltered by the quantum confinement, we performed a Fourier analysis of the QPI maps versus energy within the QCs. Fig. S2(a-d) show dI/dV maps at various voltages of the

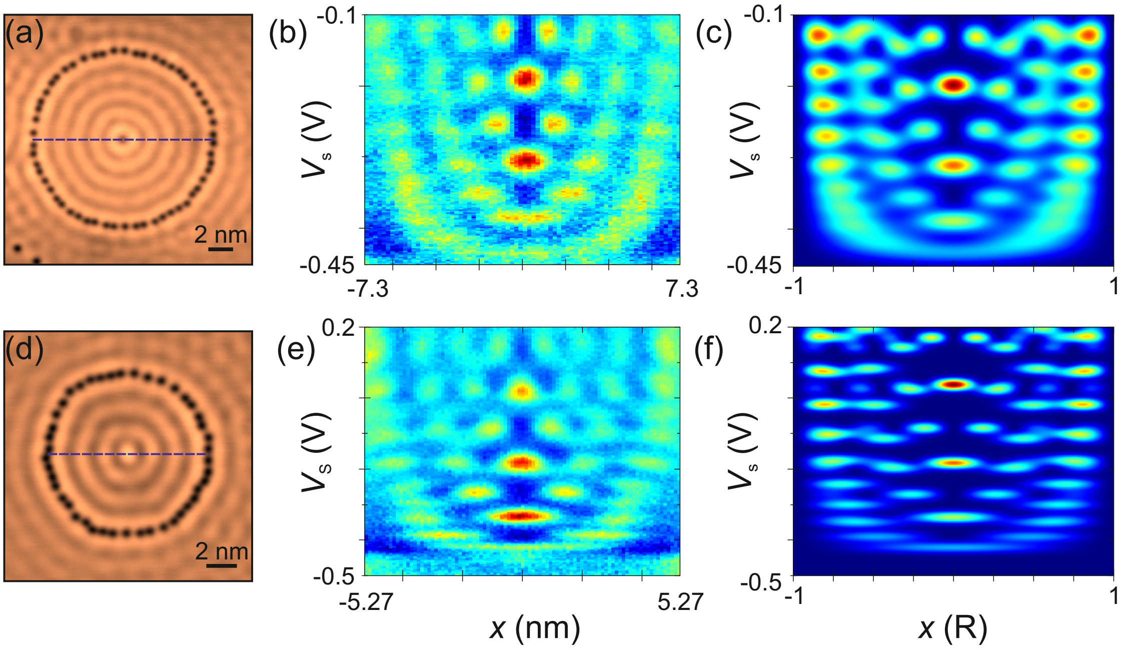

Figure S1. Constant-current STM image of a QC with radius R = 7.3 nm (V_s = 50 mV, I_t = 200 pA), formed from CO molecules on Cu(111). (b) Set of normalized STS spectra measured along the cross- sectional line indicated in (a) (V_{stab} = -0.5 V, I_{stab} = 200 pA). (c) Simulation using E_0 = -0.44 eV and m^* = 0.44 m_e. (d) Constant-current STM image of a QC with radius R = 5.27 nm (V_s = 50 mV, I_t = 200 pA) formed from CO molecules on Cu(111). (e) Set of normalized STS spectra measured along the cross-sectional line indicated in (d) (V_{stab} = -0.5 V, I_{stab} = 200 pA). (f) Simulation using E_0 = -0.44 eV and m^* = 0.44 m_e.

R = 6.15 nm QC. The corresponding Fourier transforms (FTs) are displayed in Fig. S2(e-h). Prior to the FT, images were cut to minimize contributions from areas outside the QC. Furthermore, a Welch filter was applied to minimize the influence of the Fe adatoms in all FTs.

The resulting FT images revealed a set of pronounced rings. Discussing from the center outward, first we found a pronounced circular inner ring that we attribute to the isotropic $\boldsymbol{q}_1$. This is followed by two additional rings whose shapes resemble a hexagon with rounded vertices along the $\overline{\Gamma}\overline{\mathrm{M}}$ directions (corresponding to the $\langle 100 \rangle$ directions in real space); we attribute these rings to $\boldsymbol{q}_2$ and $\boldsymbol{q}_3$, respectively. The fourth feature in the FTs is a faint and partly interrupted ring with a hexagonal shape, which we attribute to $\boldsymbol{q}_4$. Finally, we observed six outer spots that belong to the atomic lattice of $BiCu_2$, forming the $(\sqrt{3} \times \sqrt{3})R30°$ superstructure on Cu(111). In total, the standing wave patterns inside the QCs consist of four wave vectors $\boldsymbol{q}_1$-$\boldsymbol{q}_4$. For a quantitative comparison, we extracted their magnitudes along both high-symmetry directions ($\overline{\Gamma}\overline{\mathrm{M}}$ and $\overline{\Gamma}\overline{\mathrm{K}}$) for all maps acquired inside the QC with R = 7.3 nm as well as the QC with R = 6.15 nm. Their dispersive behavior is displayed in Fig. S2(i). We note that the error

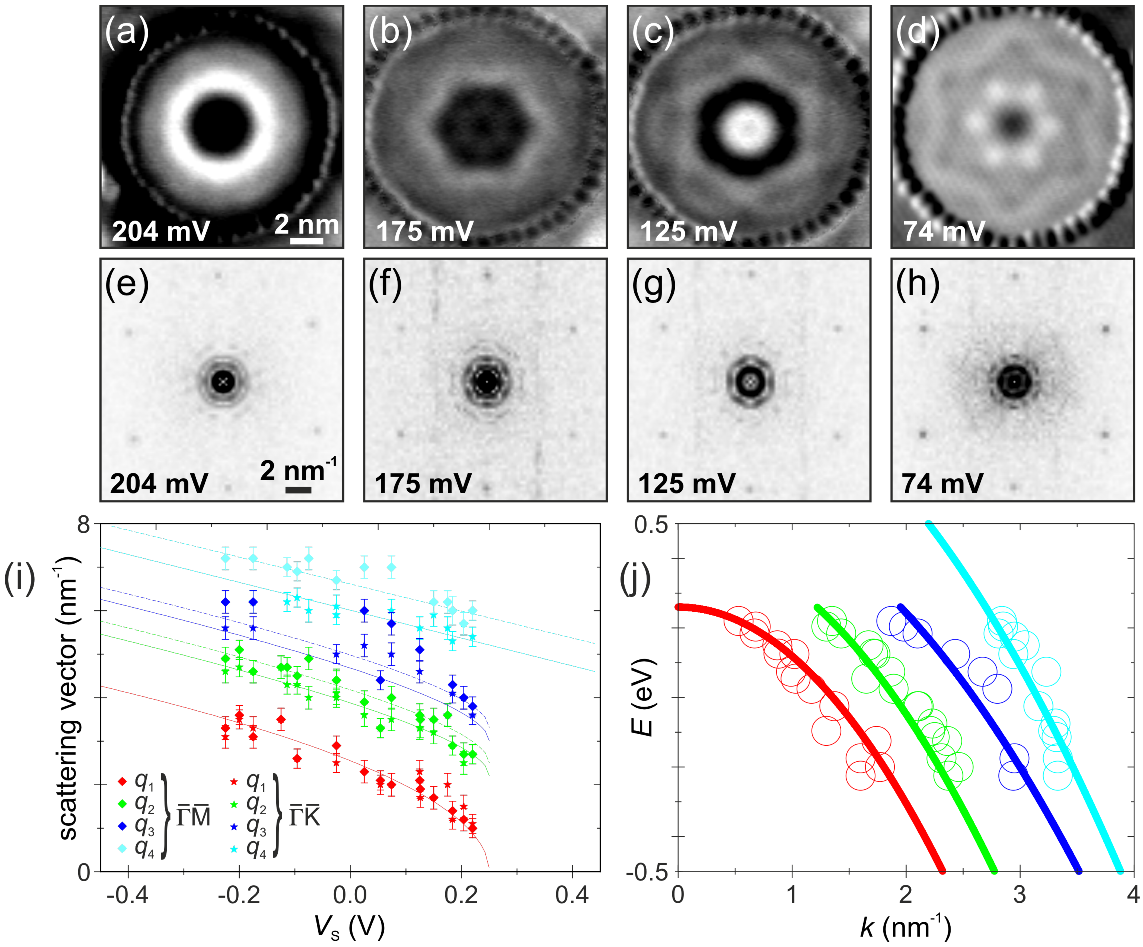

Figure S2. (a-d) Constant-current dI/dV maps and (e-h) corresponding FTs of the QC with R = 6.15 nm at various voltages (I_t = 200 pA). (i) Dispersion relation of the four scattering wavevectors obtained from an analysis of the FTs of the QCs with R = 6.15 nm and R = 7.3 nm, separately along $\bar{\Gamma}\bar{\mathrm{M}}$ and $\bar{\Gamma}\bar{\mathrm{K}}$ directions. The relatively large error bars are a consequence of the small size of the QCs. Solid (dashed) lines represent the expected dispersion relations of $\boldsymbol{q}_1$ to $\boldsymbol{q}_4$ along $\bar{\Gamma}\bar{\mathrm{K}}$ ($\bar{\Gamma}\bar{\mathrm{M}}$), based on the results from Ref. (*1*). (j) Effective isotropic parabolic bands used to simulate the possible scattering wave vectors within the band structure of $BiCu_2$, and comparison with the scattering wavevectors obtained in (i). The data points along $\bar{\Gamma}\bar{\mathrm{M}}$ and $\bar{\Gamma}\bar{\mathrm{K}}$ are averaged to obtain their mean value. The size of the data points represents the error in momentum space.

bars are due to the limited resolution in the FT image, given by the size of the QCs. Solid lines correspond to the dispersions of the four scattering wavevectors as expected from the band structure, based on the QPI FT analysis of the bare $BiCu_2$ surface by Steinbrecher *et al.*[5] As can be seen, the wave vector dispersions of the QCs are in good agreement with the literature. Also, the anisotropic character of $\boldsymbol{q}_2$-$\boldsymbol{q}_4$ is seen in our data, while $\boldsymbol{q}_1$ remains isotropic. From this, we conclude that the underlying $BiCu_2$ band structure determines the magnitudes of the possible scattering vectors within the QCs throughout the energy range probed in this study. We note that we found no difference in the

dispersions between the R = 6.15 nm QC and the R = 7.3 nm QC. Furthermore, while the dispersions of the four scattering wave vectors show good agreement both in the occupied and unoccupied states, respectively, this is not the case for the standing waves in real space, which deviate strongly from our model in the occupied states. These findings point to an energy-dependent change in the scattering potential, which varies the phase of the standing waves, not their wavelength. Due to a lack of additional input on how the scattering potential varies with energy, we did not include this in our model.

For the particle-in-a-box modelling, we took the average of the two scattering wave vectors measured for each voltage along $\overline{\Gamma}\overline{\mathrm{M}}$ and $\overline{\Gamma}\overline{\mathrm{K}}$, and we fit the dispersion of these scattering wave vectors using parabolic fits, based on the effective masses and Rashba splitting reported for the $BiCu_2$ surface.[5] We note that we did not include the hexagonal anisotropy in our model in order to be able to analytically solve the Schrödinger equation (the solutions being Bessel functions). This simplification is justified when only comparing cross sections of the spatially dependent LDOS vs. energy, because we found that the hexagonal anisotropy has a relatively small impact on the eigenstates (cf. Fig. S8). This leads to the approximated isotropic effective band structure of $BiCu_2$, shown in Fig. S2(j). These effective bands are used to simulate the confined states of $BiCu_2$. The red band stems from the inner sp_z-type Rashba-split surface state ($\boldsymbol{q}_1$), the green ($\boldsymbol{q}_2$) and dark blue ($\boldsymbol{q}_3$) bands simulate interband scattering wave vectors, and the light blue band stems from the outer p_xp_y-band ($\boldsymbol{q}_4$).[5-9] The parameters used for all QC simulations on $BiCu_2$ are E_1 = 0.26 eV, m_1 = -0.27 m_e (red); E_2 = 0.441 eV, m_2 = -0.312 m_e (green); E_3 = 0.598 eV, m_3 = -0.429 m_e (dark blue); E_4 = 0.971 eV, m_4 = -0.39 m_e (light blue).

With the parabolic dispersions, we treated each band independently using a particle-in-a-box model. The solutions are Bessel functions, which are characterized by two quantum numbers, (n,l): (n,0) and (n,1) resemble the one-dimensional particle-in-a-box solutions, with maxima and minima in the center of the QC for the (n,0) and the (n,1) states, respectively. For increasing l, the wave function becomes more localized toward the rim of the QC. We included all states present in the corresponding energy window. An additional (Gaussian) energy broadening was added, to simulate various effects of broadening (FWHM = 26 mV). We note that the experimental broadening of the states depends on the size of the QC and the state energy. For instance, comparing the peaks of the first confinement state (i.e. closest to the band onset) for the three QCs with different radius, we found widths (FWHM) of ca. 24 mV (R = 7.3 nm), 20 mV (R = 6.25 nm) and 48 mV (R = 3.1 nm), respectively. Comparing widths of peaks that are located at comparable voltages, we found widths of 26 mV (R = 7.3 nm, V_s = 174 mV),

34 mV (R = 6.15 nm, V_s = 150 mV) and 48 mV (R = 3.1 nm, V_s = 174 mV). For comparison, the experimental STS resolution of our instrument is below 2 mV. All wave functions are normalized independently to 1. To be able to weigh the contribution of each individual band, we multiplied the square of each wave function for each corresponding band with a weighting factor $w(\boldsymbol{q}_i)$. The weights were varied manually until a reasonable agreement between the model and the experiments was found. The superposition of the square of all wave functions is what is plotted in the simulated plots.

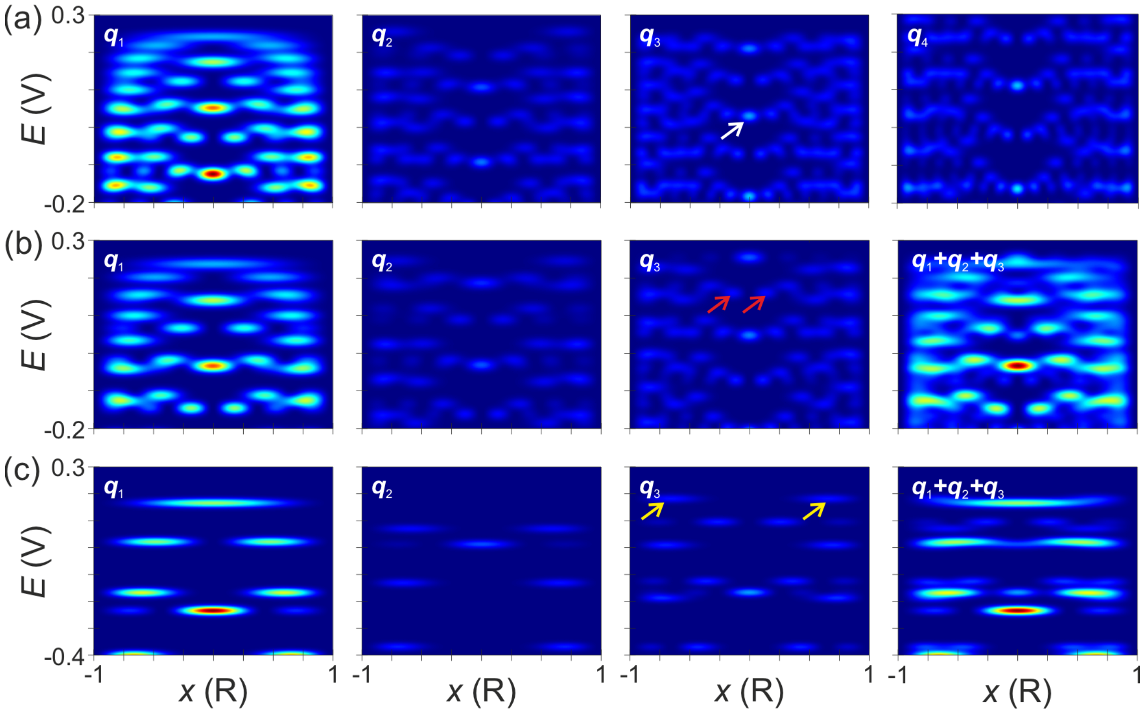

Figure S3. Disentangling the contributions to the local density of states: (a) Simulations of all effective bands present in the QC with R = 7.3 nm. The weights are $w(\boldsymbol{q}_1)$ = 5 $w(\boldsymbol{q}_2)$ = 5 $w(\boldsymbol{q}_3)$ = 5 $w(\boldsymbol{q}_4)$. (b) Simulations of the QC with R = 6.15 nm. The weights are $w(\boldsymbol{q}_1)$ = 5 $w(\boldsymbol{q}_2)$ = 5 $w(\boldsymbol{q}_3)$; $w(\boldsymbol{q}_4)$ = 0. (c) Simulations of the QC with R = 3.1 nm. The weights are $w(\boldsymbol{q}_1)$ = 5 $w(\boldsymbol{q}_2)$ = 5 $w(\boldsymbol{q}_3)$; $w(\boldsymbol{q}_4)$ = 0.

3. Disentangling the scattering wave vector contributions

To be able to attribute features present in the experimental and theoretical spectrum to a certain confined state, we plot in Fig. S3(a) the contributions of all wave vectors in the large QC (R = 7.3 nm), respectively. We use the same weights for $\boldsymbol{q}_1$- $\boldsymbol{q}_3$ as in the main manuscript. A potential contribution of $\boldsymbol{q}_4$ is included as well for the sake of completeness. The broadening in the center around 50 mV can only be explained with the confined states of $\boldsymbol{q}_3$, since it has a maximum at this energy, in contrast to $\boldsymbol{q}_2$

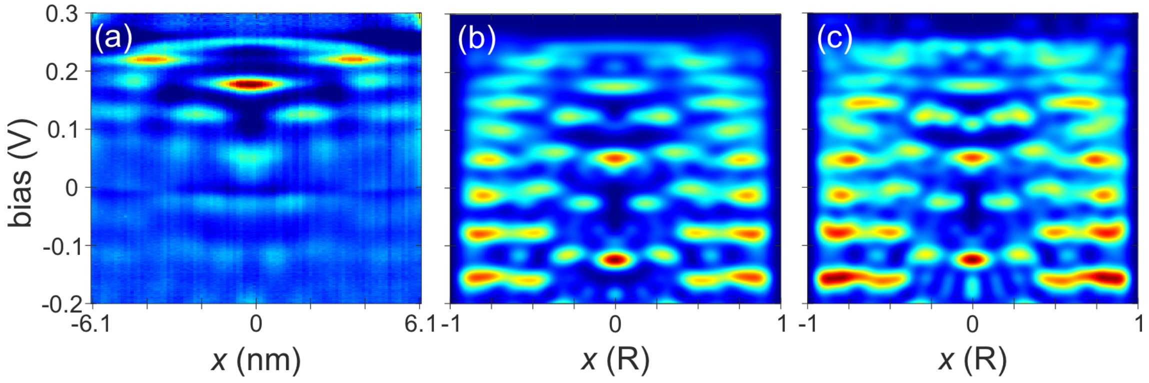

Figure S4. Including $\boldsymbol{q}_4$ to the simulation. (a) Set of normalized d*I*/d*V* spectra as presented in Fig. 3(b) of the main manuscript (V_{stab} = -0.2 V, I_{stab} = 200 pA). (b) Particle-in-a-box simulation of the QC, containing contributions from $\boldsymbol{q}_1$, $\boldsymbol{q}_2$ and $\boldsymbol{q}_3$ with fixed weights ($w(\boldsymbol{q}_1) = 5\ w(\boldsymbol{q}_2) = 5\ w(\boldsymbol{q}_3)$). (c) Simulation containing contributions from $\mathbf{q}_1$, $\boldsymbol{q}_2$, $\boldsymbol{q}_3$ and $\boldsymbol{q}_4$ with fixed weights ($w(\boldsymbol{q}_1) = 5\ w(\boldsymbol{q}_2) = 5\ w(\boldsymbol{q}_3) = 5\ w(\boldsymbol{q}_4)$).

and $\boldsymbol{q}_4$. The location is highlighted with a white arrow. Fingerprints of the confined states of $\boldsymbol{q}_3$ are found in the smaller quantum corrals as well.

Fig. S3(b) shows the contributions corresponding to the QC with R = 6.15 nm. The two red arrows point to the state that is responsible for the ring-like shape near the center of the map at 150 mV in Fig. 2(b). From the contribution of $\boldsymbol{q}_1$ alone, one would expect a maximum in the center. For the quantum corral with R = 3.1 nm, we found a ring-like state at 200 mV (see Fig. 5(a)), which is also found in the simulations of $\boldsymbol{q}_3$, see the yellow arrows in Fig. S3(c). Overall, we found multiple instances where details in the observed results can only be understood by inclusion of $\boldsymbol{q}_3$. This is very different in comparison to our calibration study using QCs on Cu(111).

Figure S4 compares the STS spectra measured across the QC with R = 7.3 nm with two simulations without (Fig. S4(b)) and with (Fig. S4(c)) the contribution of $\boldsymbol{q}_4$, respectively. Additional modulations are found in the simulation that includes $\boldsymbol{q}_4$. For example, the confined state close to the band onset (near 250 mV) shows a pronounced beating. An extra maximum is found close to 110 mV in the center of the QC. These features were, however, not found in the experiment. In addition, we did not see standing waves above the onset of the inner sp_z-type Rashba-split surface state in the experiments, where only features related to $\boldsymbol{q}_4$ could contribute. From these findings, we conclude that the impact of $\boldsymbol{q}_4$ is negligible, which is why we excluded all contributions from $\boldsymbol{q}_4$.

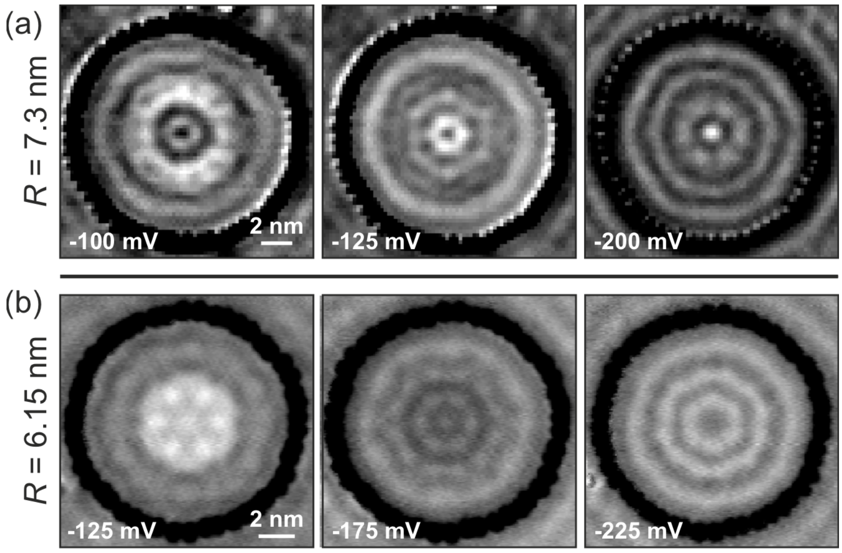

Figure S5. Confined states below the Fermi energy. (a) Constant-current d*I*/d*V* maps measured at negative bias for the QC with R = 7.3 nm (I_t = 100 pA). (b) Constant-current d*I*/d*V* maps measured at negative bias for the QC with R = 6.15 nm (I_t = 200 pA).

4. Additional maps below the Fermi energy

Fig. S5 shows additional standing wave patterns of the two QCs presented in Fig. 2, in the occupied region, where our model does not reproduce the experimental spectra. In Fig. S5(a), again an energy-dependent rotation of hexagonal features can be seen when comparing the symmetry of the maps at -125 mV and -200 mV. In comparison, this symmetry changed for the smaller QC shown in Fig. S5(b).

5. Additional electronic characterization of the smaller QCs

Fig. S6 presents complementary energy- and position-dependent LDOS data of the QCs analyzed in the main text. We start with the smallest QC we investigated in Fig. 4 of the main text, see Fig. S6(a). We show here the cross section along [110] (Fig. S6(b)), complimentary to that shown along [100] in Fig. 4(d). There was no change in the energies of the QC eigenstates when comparing both directions. The corresponding simulation for this QC is shown in Fig. S6(c) for the sake of completeness. Again, the comparability with the experiment is better above E_F, and unsatisfactory below E_F. Hexagonal anisotropies are evident in the d*I*/d*V* maps (g-i), leading to slight differences of the spatial distribution of the eigenstates (see Fig. S8).

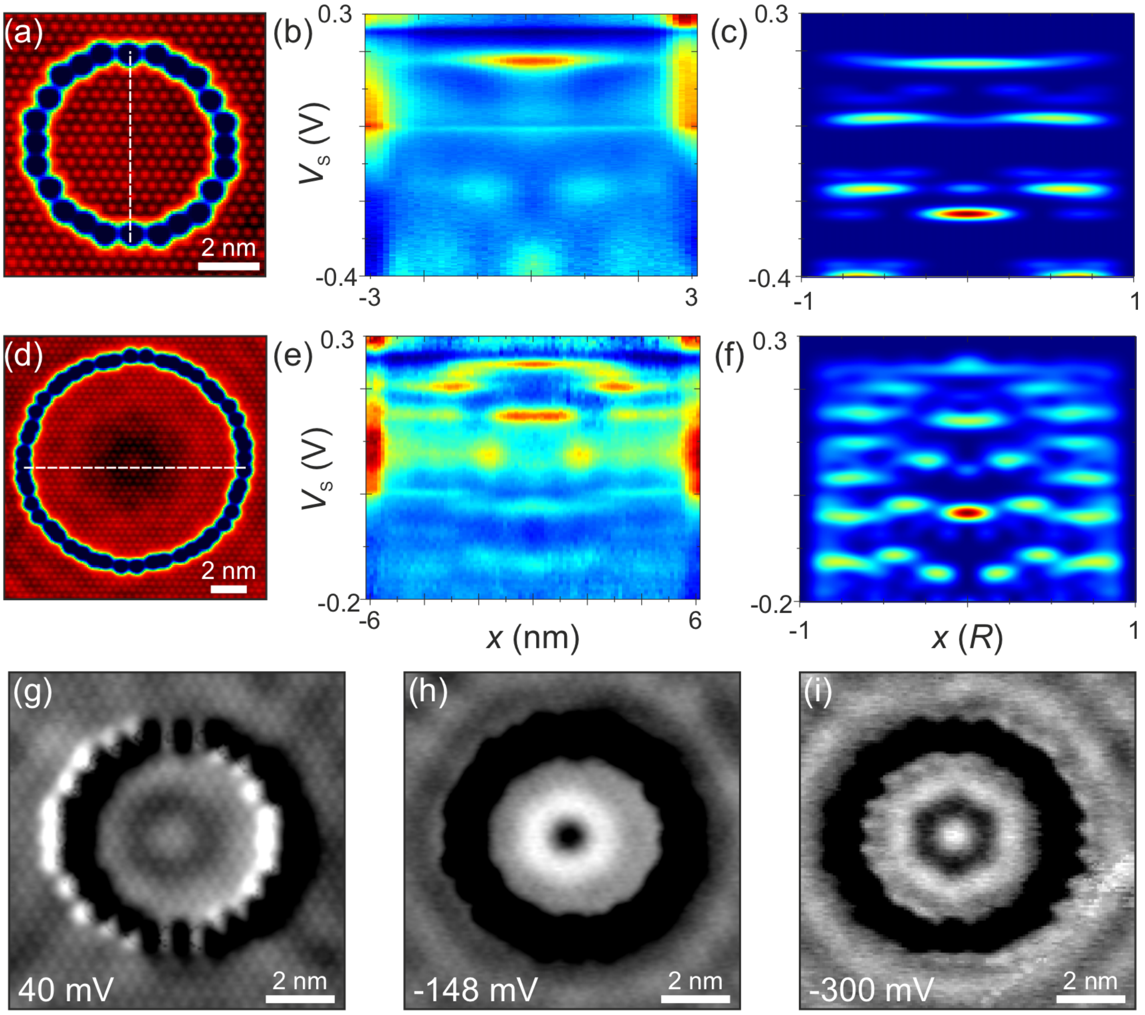

Figure S6. (a) STM image of a QC with R = 3.1nm (V_s = 5 mV, I_t = 20 pA). (b) Set of normalized dI/dV spectra measured along the line denoted in (a), which is the [110] direction (V_{stab} = -0.4 V, I_{stab} = 200 pA). A set of confined states appears inside the corral. For a comparison with the [100] direction, see Fig. S8. (c) Particle-in-a-box simulation of the QC in (a). (d) STM image of a QC with R = 6.15 nm (V_s = 5 mV, I_t = 100 pA). (e) Set of normalized dI/dV spectra measured along the line denoted in (d) (V_{stab} = -0.2 V, I_{stab} = 200 pA). Red (blue) refers to high (low) intensity. (f) Particle-in-a-box simulation of the QC in (d). (g-i) Constant-current dI/dV maps of the 3.1 QC at voltages corresponding to “state A” (g), “state B” (h) and “state C” (i) in Fig. 5 and Fig. S11(a) (I_t = 200 pA).

An STM image of the QC with R = 6.15 nm (see Fig. 2(b) in the main text for corresponding dI/dV maps) is presented in Fig. S6(d). The QC states found therein are plotted in Fig. S6(e), together with the corresponding simulation in Fig. S6(f).

To complement the dI/dV maps of the coupled QC pairs, Fig. S6(g-i) presents dI/dV maps of the QC with R = 3.1 nm at the voltages labeled state “A”, “B” and “C” in Fig. 5 and Fig. S11(a), respectively, i.e., displaying the LDOS distributions of the uncoupled QC states.

6. Ruling out artifacts in STS

All dI/dV maps presented in the main manuscript were measured in constant-current mode, i.e., the tip-sample separation was varied during data acquisition to keep the current constant. This can introduce artifacts in the data, especially when measuring close to the Fermi energy.[10] Two methods can effectively reduce these artifacts: (i) point spectra stabilized at larger bias voltage and (ii) constant height maps.[10] In case of the former, the tunneling current, given by the integrated LDOS between E_F and the applied voltage, does not vary spatially, and the measurement is essentially like a constant-height measurement.

To rule out that artifacts entered our data, we first compared the features observed in constant-current mode with maps measured in constant-height mode. Fig. S7 shows such a comparison for the QC with R = 7.3 nm. The signal-to-noise (S/N) ratio of the constant-height maps is rather low, as we had to take the map at a relatively large tip-sample separation to prevent manipulating the Fe atoms. In constant-current mode, we were able to reduce the tip-sample distance inside the QC without the risk to crash into the Fe atoms, yielding a much higher S/N. Nevertheless, all features are found in the maps for both modes. For example, we found a clear hexagonal symmetry in the constant-current map at 184 mV,

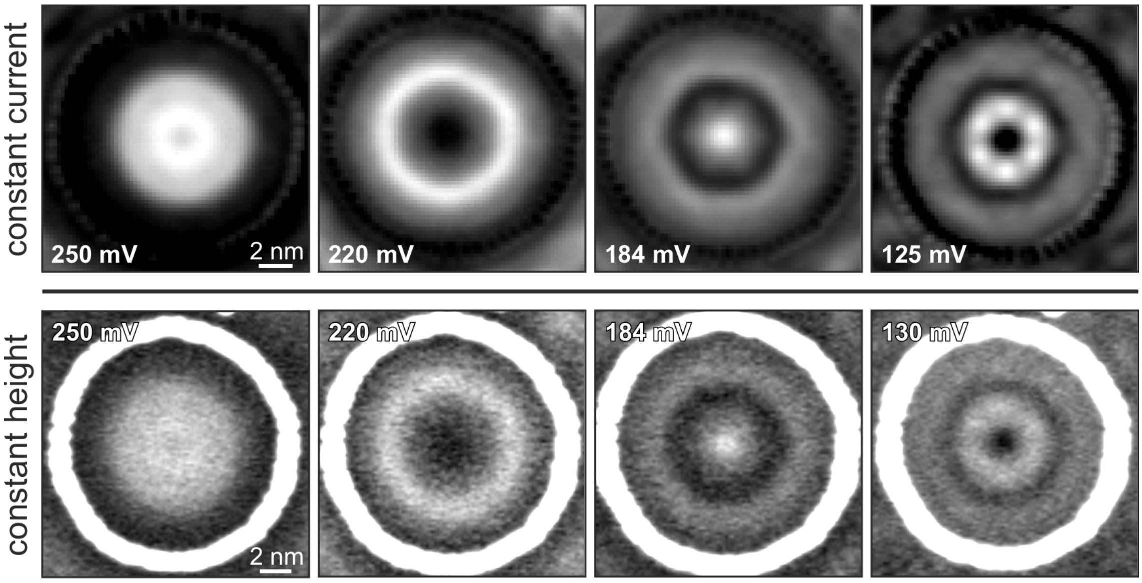

Figure S7. Comparing maps for the QC with R = 7.3 nm measured in constant-current mode (top, I_t = 100 pA) with maps obtained in constant-height mode (bottom). For the constant-height maps taken at 250 and 220 mV, the tip was stabilized above an Fe adatom with I_{stab} = 300 pA and V_{stab} = 250 mV, prior to opening the feedback and switching to the stated sample bias. For the 184-mV map, we used I_{stab} = 300 pA, V_{stab} = 184 mV. For the 130-mV map, we used I_{stab} = 400 pA, V_{stab} = 130 mV.

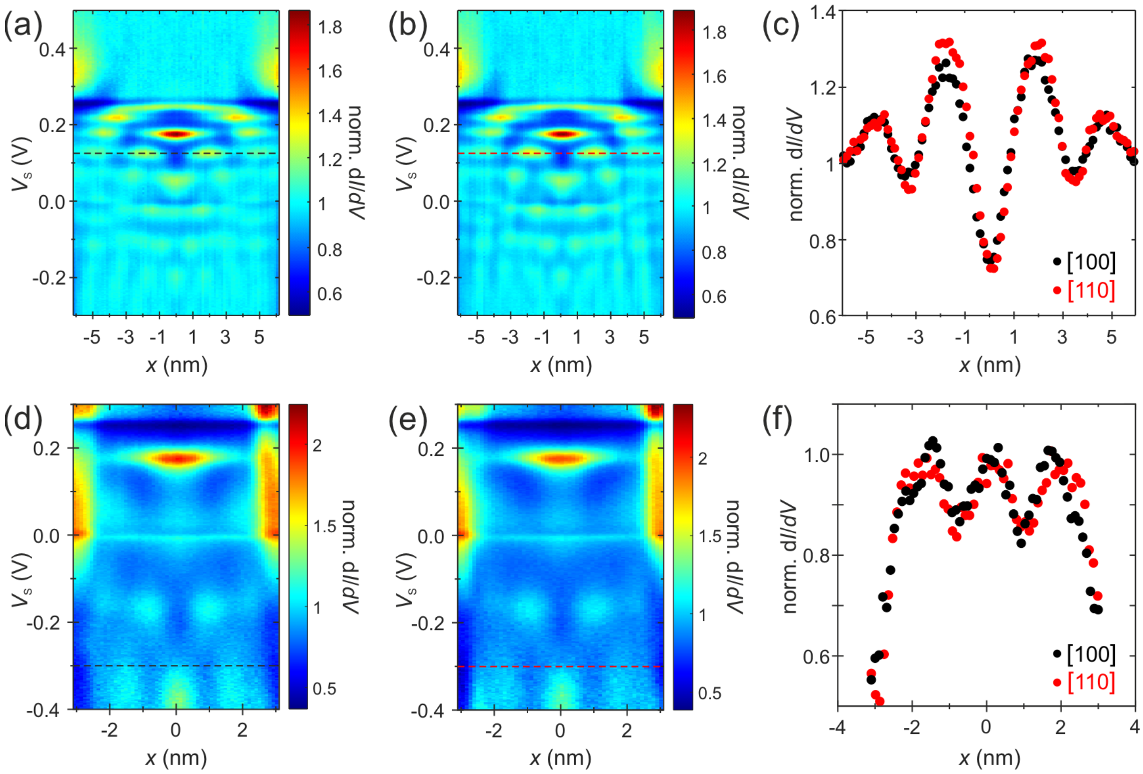

Figure S8. (a,b) Set of normalized d*I*/d*V* spectra measured across the QC with R = 7.3 nm along the [100] (a) and [110] (b) directions, respectively (I_{stab} = 200 pA, V_{stab} = 0.5 V). (c) Normalized d*I*/d*V* intensities along [100] (black) and [110] (red), as indicated by the colored dashed lines marked in (a,b). (d,e) Set of normalized d*I*/d*V* spectra measured across the QC with R = 3.1 nm along the [100] (d) and [110] (e) directions, respectively (I_{stab} = 200 pA, V_{stab} = -0.4 V). (f) Normalized d*I*/d*V* intensities along the colored dashed lines marked in (d,e).

which is also visible in the constant-height map. Also, the rotated hexagon found in the constant-current map at 125 mV is faintly visible in the constant-height map at 130 mV.

To further underline that the change in rotation of hexagonal features is not caused by artifacts of the measurement mode, we compared the constant-current map measured at 125 mV with point spectra measured across the QC with R = 7.3 nm, see Fig. S8(a-c). Prior to opening the feedback loop and acquiring the point spectra, the tip was stabilized at a high bias voltage of 500 mV to ensure that the tip-sample separation was the same at each point within the QC (i.e. the measurement is identical to a constant-height measurement). The set of spectra in Fig. S8(a) was measured along a line running horizontally across the QC (i.e. along the [100] direction), while the set in Fig. S8(b) was taken along a line running perpendicular to it (i.e. along [110]). To obtain a direct comparison, Fig. S8(c) shows the position-dependent d*I*/d*V* intensity at 125 mV (highlighted by black/red dashed lines in Fig. S8(a,b)) along both directions. While the outer maximum has similar intensity in both directions, the signal of the

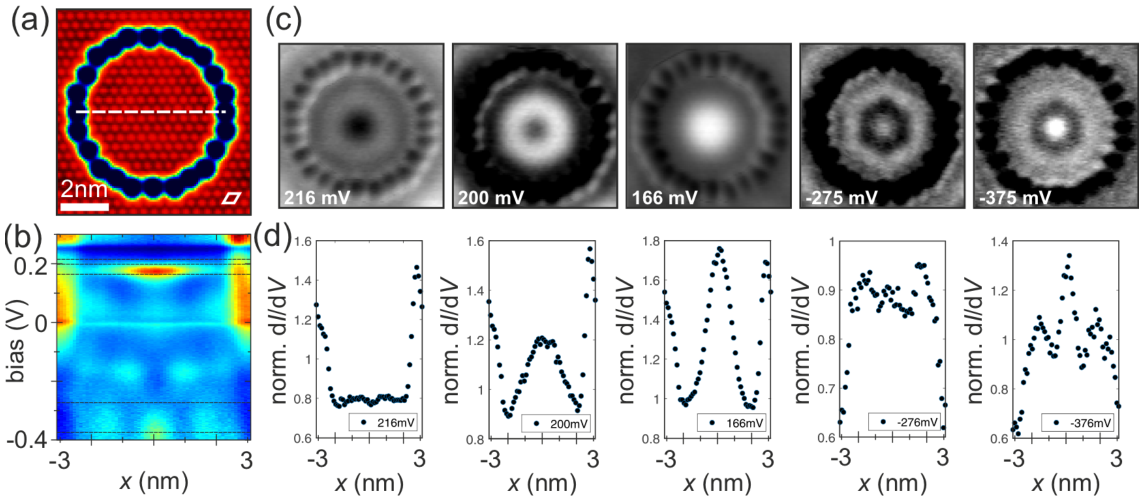

Figure S9. (a) STM image of a QC with R = 3.1 nm (V_s = 5 mV, I_t = 20 pA). (b) Set of normalized dI/dV spectra taken along the white dashed line in (a) (I_{stab} = 200 pA, V_{stab} = -0.4 V). (c) Constant-current dI/dV maps of the QC with R = 3.1 nm at various voltages (I_t = 200 pA for first three maps, and 100 pA for the last two maps). (d) Normalized dI/dV intensities along the dashed lines marked in (b) with black lines.

maxima near the center of the QC is higher along the [110] direction. These findings are fully consistent with the constant-current map in Fig. S7, in which the modulation of the inner circle has a maximum along the $\langle 110 \rangle$ directions, while it has a minimum along $\langle 100 \rangle$.

A similar direction-dependent comparison for the 3.1 nm QC is presented in Fig. S8(d-f). Here the effect of the hexagonal anisotropy shown for the linecut at -300 mV (f) clearly shows that the peripheral maximum along the [110] direction (red) is further away from the QC center, which is consistent with the dI/dV map at this energy (Fig. S6(i)) which shows hexagonal vertices along the $\langle 110 \rangle$ directions.

Finally, we compared the constant-current maps of the R = 3.1 nm QC with the spatial dependence of dI/dV intensity at the respective voltages, extracted from point spectra measured across the QC, see Fig. S9. An STM image of the QC is shown in Fig. S9(a). The dashed line across the QC represents the path of data acquisition, plotted in Fig. S9(b). To directly compare the constant-current maps in Fig. S9(c) with point spectra, we evaluated the dI/dV signal at the energies highlighted with black lines in Fig. S9(b). The results are shown in Fig. S9(d). Starting at 216 mV, we found a ring-like state inside the QC. Consistent with this finding, the dI/dV signal measured across the QC shows two maxima. At 200 mV, we found again a ring in the constant-current map. While the cross-sectional dI/dV signal of the point spectra does not reflect the node in the center, it still shows two maxima. Further analysis revealed that the dI/dV signal at 200 mV is not an eigenstate but a superposition of the ring-like state at 216 mV

and the state at 166 mV. Both these eigenstates overlap at 200 mV, due to energy broadening. The strongest dI/dV intensity was observed at 166 mV, where we found a single maximum in both the constant-current map and the point spectra. Note that the point spectra were stabilized at negative voltage (-0.4 V). Hence, the strong peak at 166 mV, which might potentially cause an artifact in the constant-current map, does not affect the point spectra. Good agreement between the two measurement modes was also found at -275 mV and -375 mV.

We note that, from a simple particle-in-a-box picture, it may be unexpected that the first eigenstate observed in the 3.1 nm QC is a ring, but this is merely a consequence of the multi-band nature of $BiCu_2$. As shown in Fig. S9(c), at $V_s \approx 0.22$ V we observe the QPI from the interband scattering channels $\boldsymbol{q}_2$ and $\boldsymbol{q}_3$, whereas there is no eigenstate from $\boldsymbol{q}_1$, yet. Contributions from the first eigenstate (i.e. an antinode in the center, which is usually referred to as the “1S” state) of $\boldsymbol{q}_1$ is observed further away from the band onset, at $V_s \approx 0.17$ V. A “1S” state of $\boldsymbol{q}_2$-$\boldsymbol{q}_4$ cannot be observed due to the dispersions of the $BiCu_2$ bands (Fig. 1(d)). As the band onset of the outer band is at a much higher energy ($E_4 \approx 1$ eV, see section S2), the “1S” state of $\boldsymbol{q}_4$ is expected to be far above the band onset ($E_1 = 0.26$ eV) of the inner Rashba-split bands. Furthermore, as the inner band does not exist above this onset, interband scattering does not exist above $V_s = 0.26$ V. This is why low q values (and hence “1S” states) do not exist for $\boldsymbol{q}_2$ and $\boldsymbol{q}_3$.

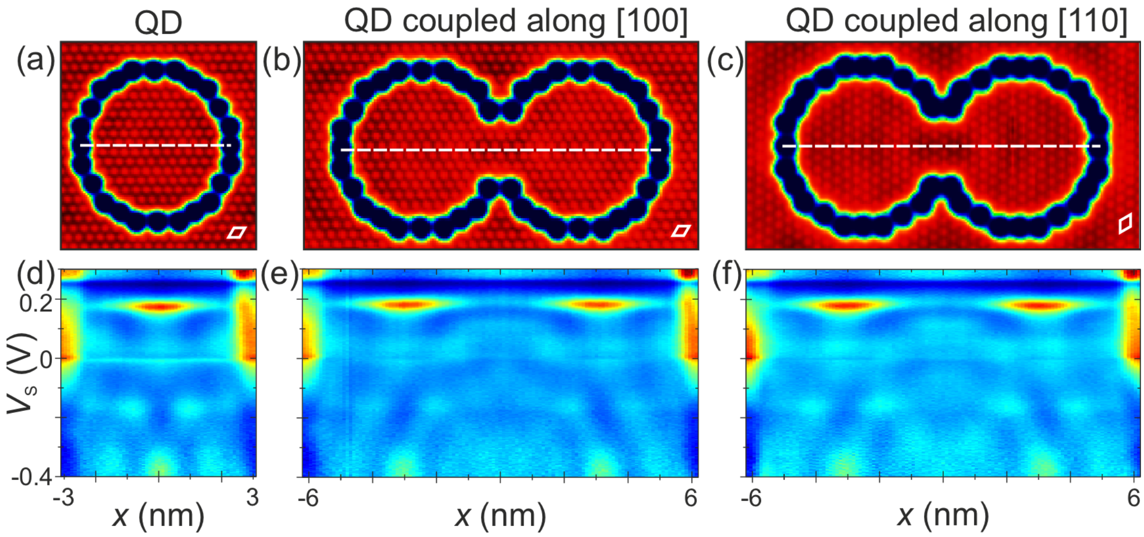

Figure S10. Full energy scale of spectra for the coupled QC pairs shown in Fig. 4. (a) STM constant-current image of the $R = 3.1$ nm QC ($V_s = 5$ mV, $I_t = 20$ pA). (b) STM image of a QC pair coupled along [100] ($V_s = 5$ mV, $I_t = 20$ pA). (c) STM image of a QC pair coupled along [110] ($V_s = 10$ mV, $I_t = 100$ pA). (d-f) Set of normalized dI/dV spectra measured along the lines denoted in (a-c), respectively ($I_{stab} = 200$ pA, $V_{stab} = -0.4$ V).

7. Additional data on coupled QC pairs

For completeness, Fig. S10 presents the same data as shown in Fig. 4 of the main text, but displayed here at the full energy range, including the dominating broad peak at $V_s \approx 0.2$ V. We could not identify a significant splitting of this peak in the coupled QC pairs. We assume that this is due the fact that this peak is due to a superposition of a hexagonal ring-like state at $V_s \approx 0.21$ V (originating from $\boldsymbol{q}_2$ and $\boldsymbol{q}_3$ scattering) and an isotopic state with a broad central antinode at $V_s \approx 0.17$ V ($\boldsymbol{q}_1$ scattering), as shown in Fig. S9. The point spectra of the single 3.1 nm QC are dominated by the 0.17 V state, but spectra taken off-center show a slight shift to higher energy by ca. 4 mV (Fig. S11(a)). For the QC pairs, an energy shift of ca. 8 mV can be seen for spectra taken at the outer edge vs. the center of the pairs (b,c). While this is indicative of a bonding/antibonding splitting, it is not reliably quantifiable for this state.

Fig. S11 provides a set of normalized d*I*/d*V* spectra taken at the center vs. the edge of the QC pairs, and comparing with those in a single 3.1 nm QC. As bonding states have their LDOS located more in junction region (i.e. the center) and antibonding states more at the outer regions of the QC pair, a direct comparison of these point spectra allows for estimating the bonding/antibonding splitting. While this effect is less obvious for the state at ca. 0.17 V (see above discussion), the splitting into bonding and antibonding states becomes obvious for states A and B, as highlighted by arrows. State C does not show clear splitting of peaks. The LDOS changes are rather subtle and only visible upon direct

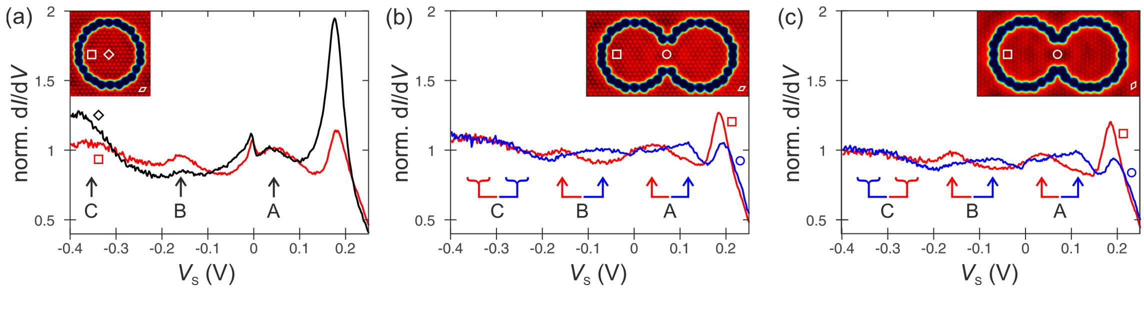

Figure S11. Identification of bonding/antibonding splitting in point spectra. (a) Normalized d*I*/d*V* spectra taken inside the $R = 3.1$ nm QC ($I_{stab} = 200$ pA, $V_{stab} = -0.4$ V). Apart from the sharp zero-bias feature, four main peaks are identified: a large peak around 0.17 eV (unlabeled) caused by a superposition of two eigenstates (see Fig. S9), as well as three eigenstates labeled A-C. (b,c) Normalized d*I*/d*V* spectra of the QC pair coupled along [100] (b) and [110] (c), respectively. The red spectra were taken at the outer edge of the QC pairs (square in the inset STM image), while the blue spectra (marked by a circle) were acquired in the center, i.e. at the junction where the two QCs are connected. The splitting into bonding and antibonding states is highlighted by arrows.

comparison of the two spectra. However, the d*I*/d*V* maps in Fig. 5 clearly show nodes and antinodes in the center of the QC pairs.

8. Structural models of the QCs

Figures S12 presents structural models of the *R* = 7.3 nm and *R* = 6.15 nm QCs, including the exact locations (i.e. adsorption sites) of the Fe adatoms on the $BiCu_2$ surface. Fig. S13 shows the same for the *R* = 3.1 nm QC as well as the QC pairs coupled along [100] and [110], respectively.

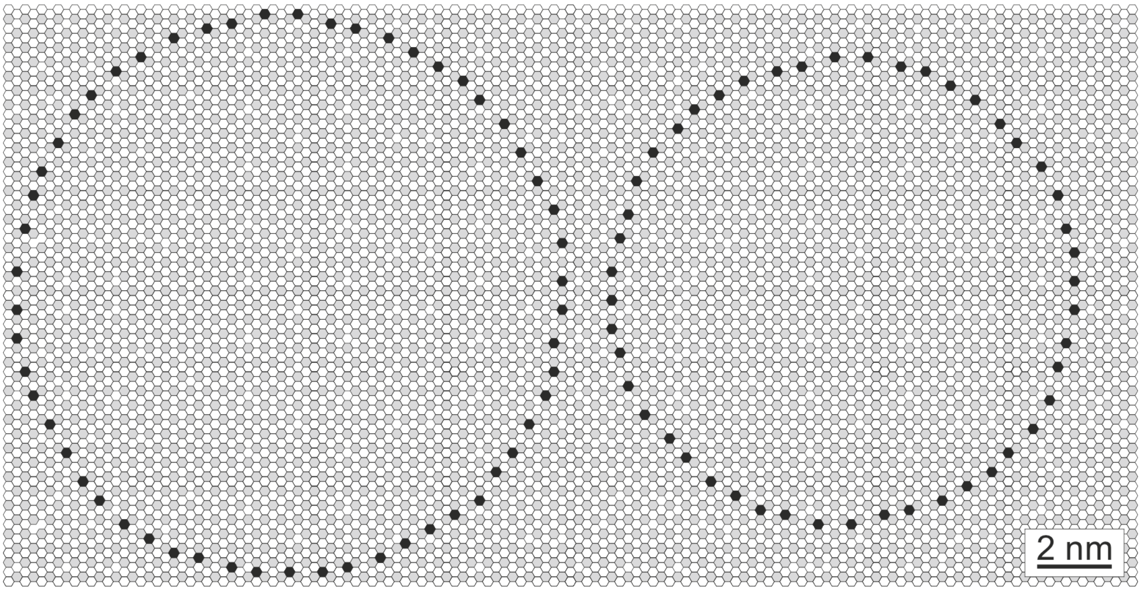

Figure S12. Structural models of the QC with *R* = 7.3 nm (left) and *R* = 6.15 nm (right). White: Cu; gray: Bi; black: Fe.

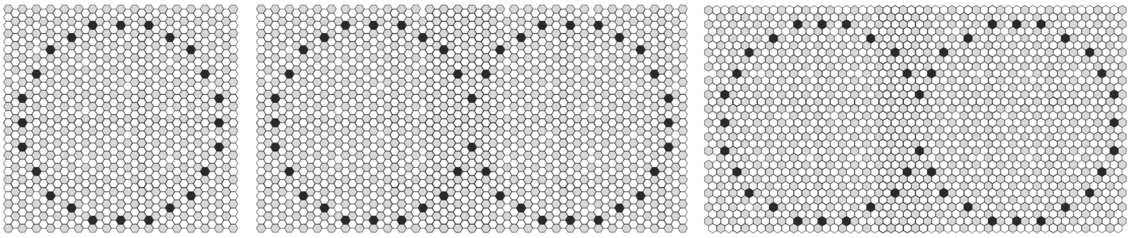

Figure S13. Structural models of the QCs with *R* = 3.1 nm: single QC (left), QC pair coupled along [100] (center) and along [110] (right). White: Cu; gray: Bi; black: Fe.

9. Maps close to the Fermi energy

Figure S14 presents two LDOS maps for the QC with R = 7.3 nm of the two confinement states closest to the Fermi energy. The map of the unoccupied state (V_s = 54 mV) qualitatively differs significantly from that of the occupied state (V_s = -25 mV), which is evidence that these are two different confinement states.

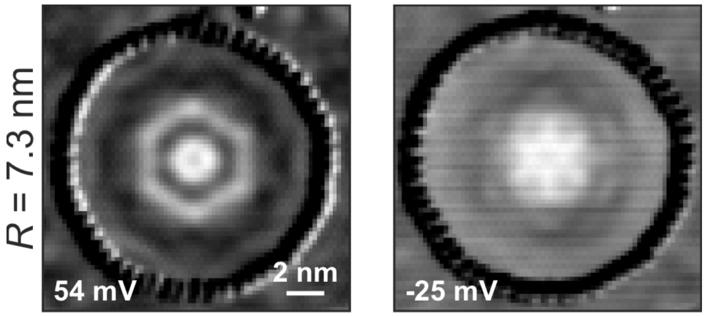

Figure S14. Constant-current dI/dV maps for the QC with R = 7.3 nm showing the two confinement states right above and below the Fermi energy, respectively (I_t = 100 pA).

References

1. Freeney, S. E.; Borman, S. T. P.; Harteveld, J. W.; Swart, I., Coupling Quantum Corrals to Form Artificial Molecules. *SciPost Phys.* **2020,** *9*, 085.
2. Crommie, M. F.; Lutz, C. P.; Eigler, D. M., Confinement of Electrons to Quantum Corrals on a Metal Surface. *Science* **1993,** *262*, 218-220.
3. Stilp, F.; Bereczuk, A.; Berwanger, J.; Mundigl, N.; Richter, K.; Giessibl, F. J., Very Weak Bonds to Artificial Atoms Formed by Quantum Corrals. *Science* **2021,** *372*, 1196-1200.
4. Slot, M. R.; Gardenier, T. S.; Jacobse, P. H.; van Miert, G. C. P.; Kempkes, S. N.; Zevenhuizen, S. J. M.; Smith, C. M.; Vanmaekelbergh, D.; Swart, I., Experimental Realization and Characterization of an Electronic Lieb Lattice. *Nat. Phys.* **2017,** *13*, 672-676.
5. Steinbrecher, M.; Harutyunyan, H.; Ast, C. R.; Wegner, D., Rashba-Type Spin Splitting from Interband Scattering in Quasiparticle Interference Maps. *Phys. Rev. B* **2013,** *87*, 245436.
6. Bentmann, H.; Forster, F.; Bihlmayer, G.; Chulkov, E. V.; Moreschini, L.; Grioni, M.; Reinert, F., Origin and Manipulation of the Rashba Splitting in Surface Alloys. *Europhys. Lett.* **2009,** *87*, 37003.
7. Bentmann, H.; Kuzumaki, T.; Bihlmayer, G.; Blügel, S.; Chulkov, E. V.; Reinert, F.; Sakamoto, K., Spin Orientation and Sign of the Rashba Splitting in Bi/Cu(111). *Phys. Rev. B* **2011,** *84*, 115426.
8. Mirhosseini, H.; Henk, J.; Ernst, A.; Ostanin, S.; Chiang, C. T.; Yu, P.; Winkelmann, A.; Kirschner, J., Unconventional Spin Topology in Surface Alloys with Rashba-Type Spin Splitting. *Phys. Rev. B* **2009,** *79*, 245428.
9. Moreschini, L.; Bendounan, A.; Bentmann, H.; Assig, M.; Kern, K.; Reinert, F.; Henk, J.; Ast, C. R.; Grioni, M., Influence of the Substrate on the Spin-Orbit Splitting in Surface Alloys on (111) Noble-Metal Surfaces. *Phys. Rev. B* **2009,** *80*, 035438.
10. Macdonald, A. J.; Tremblay-Johnston, Y. S.; Grothe, S.; Chi, S.; Dosanjh, P.; Johnston, S.; Burke, S. A., Dispersing Artifacts in FT-STS: A Comparison of Set Point Effects across Acquisition Modes. *Nanotechnology* **2016,** *27*, 414004.
